# Alzheimer's Disease: Treatment Challenges for the Future

**DOI:** 10.1111/jnc.70176

**Published:** 2025-07-31

**Authors:** John Hardy

**Affiliations:** ^1^ Department of Neurodegenerative Disease UCL Institute of Neurology United Kingdom; ^2^ UK Dementia Research Institute at UCL London UK

**Keywords:** Alzheimer's disease, amyloid, amyloid‐related imaging abnormality, biomarkers, therapy

## Abstract

The approvals of the first anti‐amyloid antibodies for the treatment of Alzheimer's disease have changed both the clinical and research landscape for the disease. These antibodies, lecanemab and donanemab, mark a turning point for our understanding of the disease pathogenesis and for the treatment of this prevalent disorder. This review discusses what they imply for disease pathogenesis and what is needed to progress from the current imperfect therapies toward safe and better, disease halting therapies. The research over the next period will involve drug development, largely aimed at reducing the side effects of the anti‐amyloid therapies, biomarker and genetic research to try and identify patients earlier in the disease process, and neuropathological research in individuals who have received treatment to try and understand the pathological substrates of the continuing clinical decline in the disease.

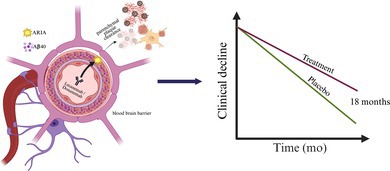

## Graphical abstract

Left hand image shows treatment antibody circulating through the brain blood vessels at high concentration where it hits the congophilic angiopathy and can induce an inflammatory reaction known as amyloid‐related imaging abnormality. A lower concentration gets through the blood brain barrier where it clears the plaque amyloid. This leads to plaque clearance and (right hand image) a slowing of clinical decline of about 25% sustained over 18 months of the clinical trial.

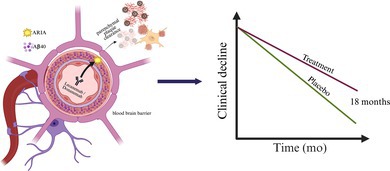

**Abbreviations**
AD: Alzheimer's disease
ARIA: amyloid related imaging abnormality
CSF: cerebrospinal fluid
MRI: magnetic resonance imaging
PET: positron emission tomography

With the approval and marketing of two anti‐amyloid antibodies (Lecanemab and Donanemab) as the first mechanistic therapies for Alzheimer's disease (AD) (van Dyck et al. 2022; Sims et al. 2023), we have clearly reached a turning point in our attempts to try and treat the disease. These drugs act by removing amyloid from the brain and differ from previous antibodies which prevented amyloid build up but did not lead to its effective removal from the brain. The issues differentiating successful antibodies are discussed in (Karran and De Strooper 2022) and the contrast between successful and unsuccessful treatments is summarized in (Hardy and Mummery 2023).

In my view, these successes are the important proof that was required to validate the amyloid hypothesis. This hypothesis has sparked a 30‐year debate about its utility (e.g., Herrup 2015; Castellani et al. 2024). Criticisms of the hypothesis centered on its undoubted oversimplicity and on the fact it was based largely on the rare early‐onset forms of disease and may not apply to the more common late‐onset cases of disease. The hypothesis was, however, always intended to provide a roadmap toward treatment and the fact that it has done so makes clear its utility. Even so, the fact that these antibodies do not, at least in their present use, stop disease may indicate that indeed there are other factors at play in the clinical decline beyond amyloid. Clearly, we need to understand the reasons for the residual decline and whether, for example, anti‐tau therapies may further slow disease progress (Mummery et al. 2023).

While these anti‐amyloid antibodies clearly slow disease progression, and therefore have passed assessment by most regulatory authorities, there is much room for improvement before we can claim we can defeat the disease. The purpose of this article is to discuss the improvements we need to make.

1. The treatments slow but do not stop the disease. The pathogenesis of the remaining decline is not clear, and understanding this is an urgent priority.
2. In a minority of patients, there is a serious effect of medication, termed amyloid‐related imaging abnormality (ARIA) which is caused by an inflammatory reaction as the antibody hits the amyloid in the blood vessel walls (Greenberg et al. 2025). APOE4 homozygotes have higher amyloid in their vessels (Schmechel et al. 1993) and this has led some jurisdictions to restrict access to the antibodies to those who are E4 homozygotes and also to restrict their use in those taking anticoagulants (e.g., https://www.ema.europa.eu/en/news/leqembi‐recommended‐treatment‐early‐alzheimers‐disease). In any event, the ARIA requires regular monitoring by MRI and regular specialist center visits, and these requirements add to the expense of the treatment.
3. The current approvals are only for those early in the disease process, and they require proof of diagnostic accuracy in terms of amyloid deposition as measured by either PET scan or by CSF biomarkers (https://www.fda.gov/news‐events/press‐announcements/fda‐converts‐novel‐alzheimers‐disease‐treatment‐traditional‐approval; https://www.fda.gov/drugs/news‐events‐human‐drugs/fda‐approves‐treatment‐adults‐alzheimers‐disease) (De Strooper et al. 2025). Additionally, the vast majority of those who received the drugs in trials were of white, northern European descent, and the utility of the drug in other populations still requires assessment.
4. The current administration regimen for the drugs is intravenous every 2–4 weeks, which also requires doctor visits. The possibility of drug “holidays” once amyloid has been removed has not yet been assessed. In addition, the safety protocols require frequent MRI assessments of ARIA. These factors mean the medical costs around their use are very high. What we do not yet know is whether, when we stop treatments with these antibodies, amyloid deposition immediately begins again or whether we can safely stop treatment for a while. Clearly, if such a drug holiday were possible, it would cut down both on the cost of the drug and the cost of safety monitoring, as well as the patient and caregiver burden.

## 1 Slow but Do Not Stop Disease

Both lecanemab and donanemab slow disease by between 25% and 30% over the period of their trials (18 months). Whether this constitutes a meaningful improvement is a topic of debate given the safety issues, monitoring burden, and expense. Additionally, we do not yet know the effects of longer‐term treatments. Clearly, we await data on patients with longer drug exposure with longitudinal assessment and pathological investigations of such patients to understand their residual pathology. That data is in process both from the open label extensions of the original trials and from the “real world” use of the drugs at some academic medical centres, particularly in the United States (Paczynski et al. 2025). In the longer term, we do not know the extent to which the residual decline depends on other pathology (e.g., tangle pathology) or could be obviated by earlier treatment which might also have the benefit of reducing the problem of ARIA caused by vessel amyloid deposits. Autopsy follow‐up of patients who have received the drugs and who have had detailed clinical follow‐up will be extremely helpful in this regard.

## 2 ARIA

It seems most likely that ARIA is caused by the amyloid antibodies initially attacking the vessel amyloid (Greenberg et al. 2025). This is outside the blood–brain barrier and so it is exposed to high concentrations of antibody, particularly after their initial infusions. ApoE4 homozygotes have generally got the highest blood vessel concentrations of amyloid and thus generally have the greatest risk of severe ARIA reactions (Schmechel et al. 1993) (strictly, these are not side effects because they are a consequence of the drug hitting its target). There are two conceptually different approaches to reducing the dangers of ARIA. The first is to give the drug before vessel amyloid buildup has begun, and the second is to tie the drug to a molecular chaperone to take the drug across the blood–brain barrier.

While giving the drug before amyloid deposition begins in the blood vessels sounds conceptually easy in the Mendelian families and in Down syndrome where the ages at the beginning of amyloid deposition are known with reasonable accuracy (Petit et al. 2022; Grigorova et al. 2022), the clinical trials necessary to assess both ARIA occurrence and clinical outcome would, of necessity, have to be several years long. In typical late‐onset disease, the additional complexity is to make predictive but accurate diagnosis in a population of individuals who you do not know whether they will get the disease. While biomarkers and genetic analyses may help in this regard, it is still a difficult problem with no easy solution even in white populations (Stevenson‐Hoare et al. 2023).

Brain shuttle approaches, where receptor‐mediated transcytosis carries the antibody across the blood–brain barrier, which means that the blood vessels can be exposed to lower antibody concentrations. The first of these drugs is trontinemab, which is a shuttle‐adapted version of gantenerumab (Grimm et al. 2023). Phase 2 data from a trial of trontinemab indeed look hopeful, with very rapid clearance of amyloid from the brain; although one fatality in this trial has to temper enthusiasm until larger trials of this approach are reported.

## 3 Increasing Diagnostic Accuracy Especially in Non‐White Populations

In white populations, the use of PET scans and of CSF Aβ and pTau measurements have considerably improved the accuracy of diagnosis of Alzheimer's disease in mildly demented white individuals. Neither approach is entirely satisfactory. PET scanning is extremely expensive and many individuals (and their physicians) blanch at CSF draws. The massively increased sensitivity of plasma biomarker analysis promises to revolutionize plasma biomarker analysis as a diagnostic aid for the disease. Plasma pTau217, (a plaque marker) which appears to be a plasma marker of neuritic plaques, efficiently separates Alzheimer cases from controls. However, its utility in separating AD from other dementias is largely untested as yet and, although it separates cases from controls, there have been few attempts to separate Alzheimer's disease from other dementias (Zetterberg and Schott 2022). Additionally, it is not yet clear how early in the disease process the separation is reliable and clearly (see above) we want to be able to make early and accurate diagnosis. This is the situation in white populations and also probably in Chinese populations (Zhong et al. 2024). However, in other, especially black populations there have been far fewer studies and the limited data available suggests that much more work is yet required to reach satisfactory diagnosis outcomes in these populations (Molina‐Henry et al. 2024).

## 4 Administration and Safety Protocols

The current drug administration require, in some jurisdictions, apoe genotyping before the drug is administered and that those who are apoe4 homozygotes as well as those who are taking anticoagulants would not be eligible. They also require that during administration there are regular assessments including MRIs to look for signs of ARIA (which, if positive, can then lead to the pausing of therapy). These processes are expensive and burdensome for both caregivers and patients as well as their clinical teams. It is possible that, as “real world data” is accrued that some of these requirements will be relaxed (e.g., ARIA is usually a problem after the first or second administration). Additionally, the current administrations are intravenous, but subcutaneous versions (which can be self‐ or caregiver administered) are being tested. This means that, as more experience of these drugs is gathered, there may be a gradual easing of the burdens their use places on patients and health care systems. A further possibility, currently being tested, is whether drug “holidays” would be possible. Once amyloid is removed, if antibody administration is stopped, how long would it be before amyloid build up starts again and clinical benefit is lost. If such holidays are possible, then this would reduce both the cost and the burden. This too is currently being tested.

## 5 Conclusion

As the discussion above indicates, although a corner in Alzheimer therapy has been turned, there is still a long road ahead in terms of getting adequate therapies to all. The road ahead, however, is largely practical rather than conceptual. And I think these therapies and their successors will continue to make inroads into the treatment of the disease. One important scientific concern relates to the underlying pathogenesis. These therapies depend on reactivating the microglia. One aspect of the disease seems to be microglial exhaustion (Streit and Xue 2014) and it will only be with long‐term drug administration we will learn whether it is possible to permanently push back the encroaching failures. This is something that only time will tell.

## Author Contributions

**John Hardy:** writing – original draft.

## Conflicts of Interest

I have consulted for Eisai, Eli Lilly, and Roche in the last 3 years.

## Peer Review

The peer review history for this article is available at https://www.webofscience.com/api/gateway/wos/peer‐review/10.1111/jnc.70176.

## Data Availability

Data sharing is not applicable to this article as no new data were created or analyzed in this study.
